# Respiratory Management in the Patient with Spinal Cord Injury

**DOI:** 10.1155/2013/168757

**Published:** 2013-09-09

**Authors:** Rita Galeiras Vázquez, Pedro Rascado Sedes, Mónica Mourelo Fariña, Antonio Montoto Marqués, M. Elena Ferreiro Velasco

**Affiliations:** ^1^Critical Care Unit, Complexo Hospitalario Universitario A Coruña, CP. 15006, A Coruña, Spain; ^2^Critical Care Unit, Complexo Hospitalario Universitario de Santiago de Compostela, CP. 15702, Santiago de Compostela, Spain; ^3^Spinal Cord Injury Unit, Complexo Hospitalario Universitario A Coruña, CP. 15006, A Coruña, Spain; ^4^Department of Medicine, University of A Coruña, CP. 15006, A Coruña, Spain

## Abstract

Spinal cord injuries (SCIs) often lead to impairment of the respiratory system and, consequently, restrictive respiratory changes. Paresis or paralysis of the respiratory muscles can lead to respiratory insufficiency, which is dependent on the level and completeness of the injury. Respiratory complications include hypoventilation, a reduction in surfactant production, mucus plugging, atelectasis, and pneumonia. Vital capacity (VC) is an indicator of overall pulmonary function; patients with severely impaired VC may require assisted ventilation. It is best to proceed with intubation under controlled circumstances rather than waiting until the condition becomes an emergency. Mechanical ventilation can adversely affect the structure and function of the diaphragm. Early tracheostomy following short orotracheal intubation is probably beneficial in selected patients. Weaning should start as soon as possible, and the best modality is progressive ventilator-free breathing (PVFB). Appropriate candidates can sometimes be freed from mechanical ventilation by electrical stimulation. Respiratory muscle training regimens may improve patients' inspiratory function following a SCI.

## 1. Background

Respiratory complications are the main cause of morbidity and mortality in the acute phase of SCI, with an incidence of 36% to 83%. Approximately two-thirds of patients with acute SCI will experience complications such as atelectasis, pneumonia, and respiratory failure, which will require mechanical ventilation [1, 2]. The degree of respiratory dysfunction is related to the extent and level of the neurological injury [3], in such a way that high cervical and thoracic injuries are at the highest risk. Various studies have suggested an increasing trend in cervical injuries, in particular, C1–C4 injuries, with an increased rate of SCI resulting in mechanical ventilation dependency [4, 5].

In SCI, respiratory dysfunction that leads to respiratory complications may be related to 3 factors: VC impairment (a reduction in respiratory muscle strength and fatigue, a reduction in inspiratory capacity, and atelectasis), retention of secretions (increased production of secretions, ineffective coughing), and autonomic dysfunction (increased secretions, bronchospasms, and pulmonary edema) [6].

Although pulmonary complications are a common and well-known problem in SCI, there is little information about their management; current practice is mainly based on clinical experience and expert opinion [7]. Promptness of prevention and treatment, as well as a multidisciplinary treatment approach by professionals experienced in the treatment of SCI, reduces respiratory complications. In this review, we provide an update on the treatment of respiratory dysfunction, the strategies for mechanical ventilation, and the criteria for tracheotomy and ventilator weaning in SCI. 

## 2. Respiratory Pathophysiology

The muscles of respiration comprise three groups: the diaphragm, the intercostal and accessory muscles, and the muscles of the abdomen. The process of inspiration involves contraction of the diaphragm and the external intercostal muscles that allow the chest cavity to expand. At high levels of ventilator activity, the accessory muscles are recruited to aid in this process. Expiration is largely passive but can be augmented by the forceful contraction of the muscles of the abdominal wall [6, 8].

The degree of respiratory failure associated with traumatic injuries to the spinal cord depends on the level of the spinal lesion. In general, functional impairment worsens as the level of injury is more rostral. In addition, a complete SCI, defined as the absence of motor or sensory function below the injury (classified as American Spinal Injury Association (ASIA) score A), results in greater functional impairment than incomplete injuries (ASIA scores B–D). Other factors that are associated with pulmonary complications are age, preexisting medical illnesses, and associated major traumatic injuries.

Individuals with SCI exhibit reduced lung volumes and flow rates as a result of respiratory muscle weakness. Changes in spirometric measurements in SCI are dependent on injury level and posture [8, 9]. Spirometric values of forced vital capacity (FVC), 1 sec forced expiratory volume (FEV1), and inspiratory capacity (IC) increase with more caudal lesions [10]. As the level of injury ascends, total lung capacity is progressively reduced. The reduction in functional residual capacity occurs at the expense of expiratory reserve volume, with a compensatory increase in residual volume. The loss of expiratory reserve volume can be explained by the denervation of the abdominal musculature and other muscles necessary for forced exhalation. Goldman et al. [11] found that the abdominal wall in tetraplegic patients is twice as compliant as in normal subjects. There is recent evidence of the benefits of a semiseated position to the weaning process of patients dependent on a respirator [12] and the effect of the seated position on lung volume and oxygenation in acute respiratory distress syndrome [13]. However, it must be noted that quadriplegics have better pulmonary mechanics in the supine position than when upright [14]. In erect postures, the abdominal contents fall forwards unopposed, and the diaphragm flattens, thus impairing the rib cage expanding mechanism of the only major respiratory muscle available in tetraplegia. The increase in vital VC in the supine position is related to the effect of gravity on the abdominal contents and a concomitant reduction in residual volume. 

The time factor is important because pulmonary function of quadriplegic patients should improve as the muscle flaccidity associated with initial phase of spinal shock converts to the spasticity of paralyzed muscles; this increase in muscle tone affects both intercostal and abdominal muscles and results in a decline in the end-expiratory volume and more effective diaphragmatic contraction.

Injury to the cervical or thoracic spinal cord affects the spinal nerves that innervate respiratory muscles. The diaphragm, the major muscle of inspiration, receives its innervation from the third, fourth, and fifth cervical spinal segments. Paralysis of diaphragmatic, intercostal, and abdominal muscles results from lesions occurring above the third cervical level and, in the absence of mechanical ventilatory support, is incompatible with life. High cervical incomplete lesions (C_2_–C_4_) or cervical lesions below C5 (C5–C8) are likely to produce paralysis, weakness, or spasticity in the muscles used to perform forced respiration. In these patients, neural control of the diaphragm is preserved, and spontaneous ventilation is possible. However, in such quadriplegic patients, respiratory function is substantially compromised, and ventilator failure can occur 4.5 ± 1.2 days after injury. 

Commonly, the respiration of patients with diaphragmatic paralysis exhibits a paradoxical movement of the abdomen: the abdominal wall retracts during inspiration and protrudes during the expiration phase. This pattern, more frequent in cervical than thoracic SCI, is the result of a lack of spinal motor activation of the external intercostals combined with the excessive compliance of the abdominal wall due to weak muscle contraction. Alterations in chest wall, lung and abdominal compliance in tetraplegia are associated with an increase in the work of breathing and may contribute to respiratory muscle fatigue.

After the initial stage of spinal shock has passed, patients with tetraplegia may develop abnormal spinal reflexes that involve the abdominal muscles. This spastic contraction reduces the elastic properties of the abdominal compartment of the respiratory system. Spastic contractions of the abdominal muscles impose a substantial load on inspiratory muscles. This additional pressure must be overcome for inspiration to occur, resulting in dyspnea [9].

The main physiologic consequence of expiratory muscle paralysis is an impaired cough. The cough reflex is preserved in cervical and upper thoracic SCI. However, the inability to cough adequately is caused by a weakness of the major muscles of expiration, resulting in an accumulation of secretions [9]. 

In acute quadriplegia, some patients develop an unexplained production of excessive and tenacious bronchial mucus. It has been speculated that the bronchial mucus hypersecretion is caused by the unopposed vagal activity, perhaps related to the initial disappearance of peripheral sympathetic nervous system tone [15]. As a result of the parasympathetic imbalance, there is bronchial spasm [16], increased vascular congestion, and decreased mucociliary activity [17, 18]. Decreased mucociliary activity is also related to mechanical ventilation [19], which is associated with the development of secretion retention. These factors predispose the patient to atelectasis, pneumonia, and potentially respiratory failure [6].

## 3. Monitoring Diaphragmatic Function

The objective of monitoring the diaphragmatic function in patients with acute SCI is to help in the decisions on managing the airway.

The two most important markers that predict the need for intubation are the level of the injury and the ASIA classification. Complete lesions above C5 require intubation in virtually 100% of cases [3, 20, 21]. In these patients, elective intubation is recommended. Urgent intubation when the patient develops respiratory distress increases the risk of neurological damage due to improper manipulation of the neck or by hypoxia [20].

In selected patients with complete cervical lesions or in those with incomplete or lower lesions, a conservative management can be performed. In these cases, the lung function should be strictly monitored. A reduction in VC to below 15 mL/kg, a maximum inspiratory pressure below −20 cm H_2_O, and the increase in pCO_2_ are markers for the need for intubation [6, 22]. Exclusive monitoring by pulse oximetry is inadequate and requires arterial gasometry or capnography [22].

In patients who are intubated, the goal is to predict the time when weaning can be started and to detect those patients that can become ventilator dependent.

The studies on phrenic nerve conduction, although essential for assessing the possibility of using diaphragmatic pacemakers, do not properly differentiate between patients who can be weaned and those who are ventilator dependent. Pathological studies do not distinguish between neuropraxia, atrophy, and axonotmesis. Similarly, normal results do not guarantee a sufficient diaphragmatic force [23]. For similar reasons, diaphragmatic fluoroscopy does not predict the possibility of weaning from the respirator and should not be used as a prognostic marker [23]. 

In recent years, interest has grown in the use of ultrasound as a noninvasive, bedside approach to evaluating diaphragm function [24, 25]. Vivier et al. [26] found that the relation between diaphragm thickness measured by inspiration and expiration using ultrasound correlated adequately with the work of breathing during noninvasive ventilation. Kim et al. [27] recently demonstrated that diaphragm dysfunction diagnosed by M-mode ultrasound predicted weaning failure. However, the use of diaphragm ultrasound in patients with acute spinal cord injury has not been prospectively studied.

As with monitoring the need for intubation, spirometry with measurement of the VC and the maximum negative inspiratory pressure are the best bedside markers for initiating weaning [6, 23, 28].

More direct measures of diaphragmatic function such as transdiaphragmatic pressure and negative inspiration force diaphragm needle electromyography are invasive and of little use in clinical practice [6, 23]. Noninvasive studies such as the estimation of diaphragm dysfunction using airway occlusion pressure during magnetic stimulation of the phrenic nerves are correlated with the duration of ventilatory support [29], but their use in clinical practice is unknown.

## 4. Ventilation Modes

To ventilate a patient with acute SCI, we must take into account the peculiarities that exclusively affect these patients.

- In high cervical and thoracic injuries, ventilation will depend almost exclusively on the functioning of the diaphragm, which will be responsible for providing 90% of the tidal volume [22].
- The loss of expiratory musculature causes an impairment in the ability to produce effective coughing, leading to the subsequent accumulation of secretions [22, 28].
- The increased production of secretions secondary to autonomic dysfunction, in addition to the above, facilitates the onset of atelectasis [18, 22, 28].

Although patients with acute traumatic SCI can usually be said to have “healthy” lungs [28], up to 60% of the patients may have associated chest trauma.

There are excellent reviews on ventilation in acute pulmonary lesions [30, 31]. We will focus here on the management of patients with acute SCI and “healthy” lungs.

In contrast to the abundance of the literature on mechanical ventilation in acute pulmonary lesions, the literature on the management of specific complications of acute SCI is very limited and of low quality.

The preservation of diaphragmatic function should be a primary objective in all patients undergoing mechanical ventilation. Diaphragmatic dysfunction is a common cause of weaning failure [32]. The consequences of this dysfunction in patients who depend almost exclusively on the diaphragm to maintain effective inspiration are clear.

Although the negative effect of mechanical ventilation on the respiratory musculature has been known for many years, the specific diaphragmatic impairment was recently reported by Vassilakopoulos [32, 33] and given the name “ventilator-induced diaphragmatic dysfunction” (VIDD).

Diaphragmatic atrophy occurs early after only 18 hours of inactivity [34], and although the pathophysiology is not well understood, it is related to an increase in muscle proteolysis [34, 35]. Diaphragmatic atrophy increases with ventilation time and causes a progressive reduction in diaphragmatic function.

VIDD has been linked to diaphragmatic inactivity caused by controlled ventilation. It has been shown in animal models that assist modes attenuate VIDD [36]. These findings have not been confirmed in humans, and no difference has been found in the onset of VIDD between patients ventilated with pressure control and those ventilated with pressure support [37]. 

Our practice is to use an assisted modality. We avoid pressure support ventilation (PSV) given the lack of evidence for a better prognosis and the risk of inadequate ventilation and exhaustion in patients with reduced respiratory reserve. The objective is to maintain some level of diaphragmatic contraction, ensuring total respiratory support. Achieving this objective requires adequate interaction between the patient and the respirator and the avoidance of asynchrony. 

Asynchrony can occur at any time in the respiratory cycle. With a perfect patient-ventilator interaction, the respirator should trigger in synchrony with the electrical impulses originating in the central nervous system [38]. Up to 25% of patients present some type of asynchrony while on mechanical ventilation [39]. Most of ventilators in use today trigger inspiration by a signal measured within the ventilator circuit The signal may be a fall in the pressure of the airway (pressure trigger) or a variation in the flow signal (flow trigger). Although it was initially believed that the flow trigger produced a better patient-respirator interaction [40], with current respirators, no differences have been found [41, 42]. 

In recent years, a new modality of ventilation has been reported: neutrally adjusted ventilator assist (NAVA) [43]. In this modality, the signal used by the respirator to deliver assistance is not the flow or the airway pressure, but rather the diaphragmatic electromyogram signal collected from electrodes placed on an esophageal catheter. Despite its promising theoretical advantages, to date there is little evidence of the superiority of NAVA compared with other ventilatory modalities [44]. 

Our practice is to use the flow trigger. We use the lowest level possible that avoids the autotrigger. In case an ineffective trigger is detected, we must rule out the presence of auto-PEEP (positive end-expiratory pressure). The need to overcome intrinsic PEEP in a patient with muscular weakness is because intrinsic PEEP is one of the most frequent causes of ineffective trigger. Lengthening the expiratory time, using bronchodilators, adding external PEEP, and reducing the sensitivity of the trigger are alternatives that can improve synchrony. 

Traditionally, the use of tidal volumes between 15 and 20 mL/kg has been recommended, with the goal of avoiding or treating atelectasis. The recommendation is based on the theory that high volumes improve the production of surfactant, prevent the collapse of the airway, promote recruitment, and are better tolerated by the patient [7, 18, 22, 28]. The clinical evidence for this recommendation is based on retrospective studies and case series.

One of the most referenced studies to justify this method for ventilating patients with SCI was published in the journal Spinal Cord in 1999 [45]. Peterson retrospectively reviewed 42 patients with SCI and found that those who were ventilated with >20 mL/kg were weaned 21 days earlier than those ventilated with <20 mL/kg (37.6 days versus 58.7 days). Several considerations preclude arriving at conclusions from this study: (1) these patients were not acute; (2) although the authors indicate that the two patient groups had similar characteristics, they do not explain the reason why they were ventilated with different volumes when their typical practice is to use high volumes; (3) they kept the endotracheal tube cuff partially deflated to allow for partial leak and to facilitate vocalization. As a result, it is difficult to assess which tidal volume was effective.

 Only one clinical trial has compared these two approaches [46]. The study, reported exclusively as a poster, included only 16 patients ventilated 2 weeks after the injury. There were no differences between ventilating with 10 mL/kg and 20 mL/kg.

To facilitate the removal of secretions, the postural drainage and manually assisted coughing techniques are considered essential. The use of mechanical devices such as intrapulmonary percussive ventilation [28] and mechanical insufflation-exsufflation (MIE) [18] has not been prospectively assessed in acute patients undergoing mechanical ventilation. Retrospective studies suggest their efficacy for reducing the number of hospitalizations in chronic patients [47] and for reducing the weaning time [18]. In a survey on the use of MIE, only 49% of the centers that responded acknowledged using the technique routinely [48].

Regarding PEEP, the standard recommendation is to use 0 cm H_2_O (ZEEP). The theory behind this recommendation is that it can increase air trapping in patients with expiratory muscle impairment. Taking into account that expiration is a passive phenomenon, it is difficult to justify this reasoning. The use of PEEP increases the residual functional capacity and may prevent the collapse and cyclic closure of alveoli, one of the causes directly related to the onset of ventilator-associated lung injury (VILI) [49]. Lacking evidence to support it, recommending the use of ZEEP no longer seems reasonable for the ventilation of patients with spinal injuries, at least in the acute phase.

Our standard practice in acute patients with SCI undergoing mechanical ventilation and who have “healthy” lungs is to use an assisted, pressure-controlled, ventilation mode, adjusted to achieve tidal volumes of 10 to 12 mL/kg with 5 to 7 cm H_2_O of PEEP, with the pressure plateau always below 30 cm H_2_O. The goal is to maintain total ventilator support, allowing the patient to initiate most of the cycles and attempting to adjust the inspiratory time to adapt it to the neural inspiratory time. We maintain this same ventilation modality once weaning has begun in the resting periods between trials of spontaneous respiration. In all cases of acute pulmonary lesion, we follow the lung protective ventilation strategy.

In the event of recurrent atelectasis that does not resolve despite assisted coughing, bronchodilators, postural drainage, and hydration, we can attempt to increase the tidal volume by 100 mL/day up to 15 to 20 mL/kg, as long as the plateau pressure is maintained below 30 cm H_2_O.

## 5. Weaning: When and How

Patients with cervical SCI have compromised respiratory functions and require mechanical ventilation based on the location and degree of the injury, both of which also affect their success in weaning from the respirator, which approaches 40% in patients with cervical injuries above C_4_, with increasing success in injuries below C_5_ [50]. The respiratory modality most commonly used in weaning is PVFB (T-tube) [51], with noninvasive mechanical ventilation and tracheotomy also playing a role. Other adjuvant treatments include the use of phrenic/diaphragmatic pacemakers in patients who do not have spontaneous breathing and drug treatments whose actual benefit is still untested.

The start of weaning and the strategy to employ are determined by the respiratory pathophysiology of the SCI, the level of the injury, and the degree of respiratory function at the time the weaning is started. Therefore, the patient's respiratory function needs to be assessed before and during weaning [52]. As stated previously, the best parameters for its assessment are derived from arterial gasometry or capnography, lung function tests VC, and effectiveness of cough and diaphragmatic electromyography (which is not very useful in clinical practice) [23]. 

Before the start of weaning, it is advisable to optimize breathing through the aspiration of tracheal secretions, placement of the patient in the supine or Trendelenburg position, and after the administration of bronchodilators [53]. 

Once the patient's breathing has been optimized, we assess the various weaning modalities. The three general approaches to weaning are: progressive ventilator-free breathing (PVFB) or T-tube, pressure support (PS), and synchronized intermittent mandatory ventilation (SIMV) [54]. When comparing these three weaning modalities, it has been observed that weaning patients from mechanical ventilation takes longer when using SIMV and does not improve the success rate; therefore, currently SIMV would not be indicated [55, 56]. Several studies have shown greater success with the T-tube [51, 55]. In a recent study performed on patients with prolonged mechanical ventilation, Jubran et al. [57] concluded that weaning by PVFB through tracheostomy is faster compared with SP, without affecting 6–12 month survival. When assessing this study in terms of patients with cervical SCI, it is important to take into account that one of the exclusion criteria is a bilateral phrenic injury. 

PVFB weaning consists of a respirator-free time that is gradually increased and achieves an increase in muscle force in patients with high and low cervical injuries. The procedure is started with a FiO_2_ of 10% above the respirator baseline and with only 5 minutes of disconnection per hour, which is gradually increased throughout the day depending on the patient's degree of tolerance, thereby avoiding exhaustion. The intervals of connection to the respirator therefore must be sufficient for the diaphragm to recover before the next test (approximately 2 hours). The withdrawal of mechanical ventilation can be proposed when the patient tolerates 48 hours without respiratory support. 

Regardless of the weaning modality, all studies have observed that the time for the withdrawal of the respirator in patients with SCI ranges from weeks to months [58, 59].

Thus, we will consider extubation when, after 48 hours without ventilator support, the patient meets several requirements, with one of the most important being the ability to generate a flow peak of cough > 2.71/s or a negative inspiratory pressure <−20 cm H_2_O [60]. 

*Checks before Extubation*

- There are no required surgical or X-ray diagnostic procedures close to extubation that require sedation.
- No sedative drugs, cooperative.
- Afebrile and with stable vital signs.
- Saturation > 95% and pCO_2_ < 40–45 mmHg, after >12 h breathing ambient air.
- FiO_2_ no more than 25% and PEEP < 5 cm H_2_O.
- X-rays with no abnormalities or an obvious improvement.
- Few bronchial secretions.
- Negative inspiratory pressure <−20 cm H_2_O.
- Vital capacity > 10–15 mL/kg of ideal weight.
- A normal fluid balance.
- No contraindications for performing physical therapy (fractured ribs, etc.) or for the use of noninvasive mechanical ventilation (facial fractures, etc.)

### 5.1. The Role of Noninvasive Mechanical Ventilation (NIMV)

In recent years, the use of NIMV has been proposed for patients with SCI as respiratory support both in acute conditions and in respirator weaning and as a long-term night support for patients in whom hypoventilation is detected. To use NIMV, the patient must be cooperative, have an intact bulbar musculature, and be medically stable. 

Bach and Saporito [60, 61] and Tromans et al. [62] describe the use of NIMV in acute conditions to avoid connecting patients to respirators who have a VC < 50% of its normal value; if the VC falls below 1200 mL, continuous support will be necessary. We must take into account that both studies are retrospective and had a limited number of cases; studies are therefore needed to support the use of NIMV as a substitute for invasive mechanical ventilation. NIMV may aid in respirator weaning and prevent postextubation failure [63, 64], thereby reducing reintubation-related complications. If rehabilitation is included with NIMV support, the rate of success increases considerably. Chronic SCI with reduced VC may benefit from support from nighttime NIMV in patients in whom nighttime saturation is repeatedly <95% and CO_2_ > 50 cm H_2_O, which unequivocally indicates hypoventilation. In these patients with chronic hypoventilation, nighttime oximetry monitoring, capnography, indicates to us the need for nighttime NIMV and the patients who will benefit from respiratory electrostimulation. 

NIMV can be used with two ventilator modalities: continuous positive airway pressure (CPAP), where a continuous inspiratory pressure is provided, and BPAP, which provides support through two pressures (inspiratory and expiratory), BPAP being more advantageous than CPAP because it keeps the alveoli open by providing a minimal PEEP [62]. Both modalities may be used with or without supplemental oxygen and with an interface that can be nasal, oral or nasooral. If, after administering BPAP, the VC presents a reduction >25%, weaning is discontinued. The method consists of reducing the support pressure (IPAP) in ranges of 2 cm H_2_O for 12–24 hours, going back to the start until the patient is completely weaned. The EPAP should not exceed 4 cm H_2_O during the weaning process.

One of the difficulties in the use of NIMV is that although the patient may have functionally preserved bulbar musculature innervation, NIMV may not provide sufficient force to hold the nasal or oral piece in place. Other potential complications include aerophagia, abdominal distension, and barotrauma (very uncommon). 

### 5.2. Respiratory Electrostimulation: Phrenic and Diaphragmatic Pacemakers

Phrenic or diaphragmatic pacemakers, whose use started in the 70s by Glenn et al. [65], are one of the forms of respiratory support that may be used in the withdrawal of the volumetric respirator as an alternative to long-term respiration in patients with SCI, thereby improving their quality of life. Electrostimulation of the phrenic nerve consists of triggering diaphragmatic contractions through direct electrical stimulation of the phrenic nerve in the neck and chest. Candidates for electrostimulation include patients with spinal injuries above C_4_, with functional phrenic nerves and diaphragm, and with no severe airway disease or pulmonary parenchyma. It is therefore necessary, prior to implantation, to perform phrenic nerve conduction with bilateral, simultaneous diaphragmatic fluoroscopy. After its placement using thoracotomy, a period of conditioning is needed in order to improve the muscle tone of the diaphragm, which may have atrophied due to lack of use. The daily duration of the electrophrenic respiration may be 16 hours to 24 hours, with nighttime ventilator support to support physiological recovery of the diaphragmatic muscle, in anticipation of the next day. Hirschfeld et al. [66], when comparing it to conventional ventilation, found a lower cost due to fewer episodes of infection. Romero-Ganuza et al. [67] observed greater survival with a better quality of life due to improved sociability, convenience, and mobility.

External diaphragmatic pacemakers are a modality that is more applicable to the ICU [68]. This system uses electrodes that are implanted directly using endoscopy within the diaphragmatic muscle and which are connected to an external stimulator implanted in the skin. This system can be used early on for the management of weaning in the ICU, with less likelihood of complications by avoiding the need for major surgery.

In regards to tetraplegic patients who have inadequate functionality of the phrenic nerves and in whom the implantation of phrenic/diaphragmatic pacemakers is not possible, there are experimental studies in animals that attempt to restore ventilation without respirator with high-frequency spinal cord stimulation (HF-SCS) of the intercostal muscles. DiMarco and Kowalski [69], in a study conducted on dogs, achieved periods of up to 6 hours free of respirator without muscle fatigue.

In some groups, approximately 40% of the individuals are supported at all times with these devices. It is important to note that the diaphragmatic pacemakers only supplement the inspiratory function and do not replace the expiratory functions such as coughing or the removal of secretions. It is here where rehabilitation and external mechanical assistance have considerable importance.

It is known that patients with cervical SCI have increased resistance in the airways due to a loss of sympathetic control. For tetraplegic patients, there are studies with drugs that attempt to counteract this effect, in order to facilitate weaning. In terms of drugs, we have bronchodilators (salmeterol), which appear to improve respiratory function, inducing an anabolic effect on the respiratory musculature [70]; methylxanthines (theophylline) [71], which may be effective in facilitating respirator weaning in tetraplegic patients by improving muscle force; and testosterone derivatives, which increase muscle mass and force [72]. All of these have had conflicting results.

## 6. Tracheostomy

Tracheostomy is a common procedure in patients with traumatic SCI, especially in cervical SCI or thoracic level with associated injuries. In patients with cervical SCI, tracheostomy is only one component of effective therapy. Equally essential is early intubation of those patients. For patients with incomplete injuries, evidence of respiratory failure should prompt immediate airway intervention, half of whom will require tracheostomy [73]. Many studies [3, 74–76] have tried to identify several risk factors for postinjury tracheostomy: rostral ASIA A level (C2–C4), age (>45 years), comorbid lung diseases, smoking history, preexisting comorbid illness, the severity of impaired consciousness, high level injury severity score, and active pneumonia. Specific to a cervical SCI cohort, the extent of injury was the most important factor in determining the need for a tracheostomy [77]. In a retrospective study [78], patients with high-thoracic SCI have more respiratory complications (including the need for tracheostomy), even after adjustment for age, sex, and Glasgow Coma Scale, compared with patients with low-thoracic SCI or thoracolumbar fractures. In this study, respiratory complications significantly increased the mortality risk in less severely injured patients. Winslow et al. [79] noted that respiratory complications are as important as the level of injury in contributing to the prediction of length of stay.

The literature has suggested that early insertion of a tracheostomy facilitates pulmonary management and an earlier discharge from the intensive care unit (ICU). In a retrospective study [80], early tracheostomy (<day 7) in traumatic SCI patients was associated with a shorter duration of mechanical ventilation, shorter length of ICU stay, and decreased laryngotracheal complications, but they could not demonstrate that early placement prevents the risk of ventilator-associated pneumonia or affects the mortality rate. A retrospective review of patients with cervical SCI [81] verified that tracheostomy decreased the mortality rate and pulmonary complications compared with endotracheal intubation longer than 7 days, and they demonstrated that early tracheostomy facilitates quicker extubation and shorter hospital stays. They recommend early tracheostomy if at least two of the following three factors are present: the Injury Severity Score (ISS) > 32, the patient has a complete SCI, or PaO_2_/FiO_2_ ratio <300 3 days after mechanical ventilation was initiated. Sims and Berger [82] suggest that early tracheostomy be considered in hospitalized trauma patients requiring halo fixation and who present with a high ISS, a history of cardiac disease, or a condition requiring intubation on arrival.

Both surgical and percutaneous tracheostomies can be safely performed in the ICU. Nun et al. [83] have found percutaneous tracheostomy (PT) to be a feasible and safe procedure in patients with cervical spine fracture and available anatomical landmarks without neck extension. PT is a much quicker method, minimizes injury to the adjacent neck structures, and probably has fewer late infections of the stoma [84], which could be an important advantage in patients who have undergone anterior cervical spine fixation and who require prolonged ventilator support [85]. Currently, there is a paucity of retrospective studies [50, 86–88] evaluating the period of time that should separate these procedures to determine the safety of early tracheostomy after anterior cervical spine fixation, but a period of 1-2 weeks is often used. The long-term sequelae of lateral tracheostomy in patients with anterior cervical fusion await further investigation [89].

Tracheostomy appears to facilitate weaning by decreasing the work of breathing, especially in patients with limited respiratory reserve [51]. Compared to the native airway, the tracheotomy cannula constitutes an additional resistive component. In a physiological study performed on difficult-to-wean patients, Ceriana et al. [90] found that diaphragmatic effort is lower during a T-piece trial with a deflated cuff than when the cuff is inflated. Other authors [91] have recently shown that deflated cuff during weaning strategies not only improves the time to decannulation but also decreases respiratory infections and improves swallowing function.

## 7. Conservative Management of Respiratory Dysfunction

Respiratory complications are the main cause of morbidity and mortality in the acute phase of SCI, with an incidence of 36% to 83% [1, 2]. Although pulmonary complications are a common and well-known problem in SCI, there is little information about their management; current practice is mainly based on clinical experience and expert opinion [7]. 

The pillars of early treatment of respiratory dysfunction in SCI are intensive management of secretions and atelectasis, which has been shown to improve the results in patients with SCI [28]. The most important objective of treatment is the expansion of the lungs and the clearing of secretions. The techniques commonly used to help remove secretions include assisted coughing, percussion, vibrations, aspiration, and assisted postural drainage. To increase ventilation, respiratory exercises can be used for muscle training, noninvasive positive air pressure support and high tidal volumes in patients on mechanical ventilation [92].

Interventions for mobilizing secretions are essential for preventing mucus plugs, atelectasis, pneumonia, and respiratory failure and should be started early after the injury. For best results when using respiratory therapy techniques, it is essential to have proper pain treatment to facilitate patient cooperation. Bronchospasms are continuous in patients with cervical SCI due to autonomic changes in the acute phase, with a predominance of vagus nerve tone. Bronchospasms respond well to beta-2 agonists and inhaled anticholinergic agents [93], with beneficial effects on FEV1, FVC, and PEF [94]. Their use is widely recommended and should be started before bronchospasms are apparent [6, 92] There is no question that bronchoscopy with alveolar lavage is an effective method for removing secretions, but it is not exempt from complications such as tracheobronchial irritation, edema, and aspiration. The technique should be reserved for patients who do not respond to more conservative measures or for taking microbiological samples. The multidisciplinary treatment approach for professionals experienced in the treatment of SCI reduces respiratory complications and the need for performing fibrobronchoscopy [18, 28].

### 7.1. Postural Drainage

If the patient is immobilized, postural drainage and passive positioning techniques using gravity can facilitate the movement of secretions. The goal is to move the secretions from the most peripheral regions of the lungs to the main airway, where the secretions can be more easily removed using coughing or other methods of aspiration. The positioning of the patient with the affected lung area in the upper position allows for gravity to help in the drainage. Each position (Trendelenburg, supine, prone, and left and right lateral) should be held for at least 5 to 10 minutes, depending on tolerance.

### 7.2. Percussion and Vibration

These consist of external manipulations of the chest to mobilize secretions. Percussion consists of rhythmically tapping on different areas of the chest with a cupped hand. The floating ribs should be avoided, and the intensity and duration of the percussion should be adjusted to the patient's comfort level. Vibration consists of the application of vibration with the hands to the chest wall and soft tissues of the chest during the expiratory phase. The techniques may be combined with postural drainage. Their contraindications need to be taken into account and include instability of the cardiovascular system, tension pneumothorax, pulmonary embolism, significant pleural effusion, unstable spine, contraindication for increased intracranial pressure, acute hemoptysis, fractured ribs, and chest burns and wounds. 

There are currently no studies that have demonstrated their efficacy in tetraplegic patients [7].

### 7.3. Assisted Coughing Techniques

The goal of these techniques is to help generate effective cough strength. They are often used with postural drainage, IPPB, and insufflator. Several techniques are used as follows.

#### 7.3.1. Manually Assisted Coughing

This maneuver consists of chest compressions coordinated with the patient's breathing. This attempts to imitate the normal cough, helping to move secretions from the lowest areas of the lungs. The therapist who performs the technique places the palm of the hand below the patient's rib cage, between the xiphoid process and the navel, exerting pressure upwards and inwards in sequence with the patient's voluntary expiration or cough. The external pressure acts in the place of the paralyzed intercostal and abdominal muscles. Its effectiveness can be improved with the prior administration of nebulized saline to thin the secretions. The cough can also be increased with prior lung insufflation; it is possible to generate higher expiratory flows by using larger lung volumes. It is contraindicated in cases of unstable spine in traction, internal abdominal complications, rib fractures, and recent placement of a vena cava filter.

#### 7.3.2. Mechanically Assisted Coughing (Mechanical Insufflation-Exsufflation)

This procedure is started by applying positive pressure to the airway (insufflation) using a mechanical device (Cough-Assist) to immediately afterwards transform this positive pressure into negative pressure *(exsufflation)*. This sudden change of pressure in a short period of time (<0.02 s) generates an air flow capable of pulling respiratory secretions to the exterior. Each session consists of 6–8 cycles (1 cycle usually consists of 2 seconds of *insufflation* and 3 seconds of *exsufflation*) with pressures approximately ±40 cm H_2_O, generating an expiratory flow of approximately 10 L/s, which is followed by 5–10 minutes of rest to avoid hyperventilation. Pressures below ±30 cm H_2_O are ineffective [95]. The technique can be applied through tracheotomy, facemask, or mouthpiece, and it is more effective than manually assisted coughing and reduces the need for deep endotracheal aspiration, which makes the technique less irritating for the lungs and more comfortable for the patient. The use of an insufflation-exsufflation device during intensive and postintensive care may reduce the number of bronchoscopies, the number of respiratory complications, and the weaning time [96]. Its contraindications include a history of barotrauma and the presence of pulmonary bullae.

### 7.4. Respiratory Muscle Training (RMT)

There is no clear evidence of the benefit of RMT in patients with SCI. In a systematic review of 8 studies (3 randomized, controlled clinical trials), Sheel et al. concluded that there is only level 4 evidence to support the use of inspiratory muscle training (IMT) in improving the respiratory function of patients with SCI [97]. Recently, Mueller et al. in a randomized, controlled clinical trial compared various RMT methods in tetraplegic patients. They concluded that training of the inspiratory force is more beneficial than training of the respiratory muscle resistance in improving respiratory function, voice, chest mobility, and quality of life in patients with tetraplegic during the first year of the injury [98].

IMT consists of the use of resistance during inspiration to improve the strength and resistance of the inspiratory musculature that is unaffected (or partially affected) by the SCI. The technique uses devices with spring-loaded valves that allow for expiration and offer resistance to inspiration. These devices can be connected to the tracheotomy tube or to a mouthpiece. Weights placed on the abdomen or incentive spirometers can also be used in IMT to offer resistance to inspiration. A typical IMT program requires the patient to generate an additional inspiratory pressure for 15–30 minutes 2-3 times a day [99]. The optimum inspiratory pressures are not yet known, although excessive pressures may cause respiratory fatigue and hypercapnia [100].

With regard to the expiratory musculature, the strengthening of the pectoralis major muscle may be useful (C5–C7 innervation, its function may be partially preserved), because the clavicular part helps expiration. Given that the VC in high SCI is influenced by the position of the patient (the diaphragm tends to remain partially collapsed due to the effect of gravity), it may help to place an abdominal girdle, which can improve respiratory function in the sitting position by placing the diaphragm in a more efficient position. In addition, electrical surface stimulation directly on the abdominal muscles or applied to the lower thoracic medulla can improve coughing and expiratory muscle function [100].

Although there is scant evidence from the physiological point of view, it does make sense to use RMT, at least in the ventilator weaning phase. The treatment is started only when the patient is able to tolerate periods of spontaneous respiration. Initially, the resistance is set to 7–10 cm H_2_O for a maximum of 1 minute, twice a day. The resistance, frequency and duration are gradually increased as the inspiratory force improves [101].

### 7.5. Noninvasive Ventilatory Support

Usually, this type of support is used to provide positive pressure in the airway during expiration and/or inspiration. There are various types of positive-pressure support, but the most widely used are CPAP, BPAP, and IPPB (intermittent positive-pressure breathing). Although this type of NIMV may occasionally be used to ventilate patients with high SCIs, it is typically used in respiratory weaning and as a long-term night support for patients with hypoventilation, as discussed above.

The IPPB mode can be administered with mechanical devices or with an Ambu bag. It is useful for lung expansion by introducing large volumes of air. The pressure level should be started at 10–15 cm H_2_O and gradually increased, never exceeding 40 cm H_2_O [7], It is a NIMV method rarely used in patients with SCI; however, it may be an alternative to tracheostomy in patients with high tetraplegia. It requires that the patient be conscious and cooperative. The preferred method for ventilatory support is administration through a mouthpiece during the day and through a nose mask during the night. 

Noninvasive ventilation can also be supplied through negative-pressure body ventilators such as the iron lung, the pneumobelt, and other devices [102]. They are very rarely used; however, they may provide some advantage as an option for temporary or permanent ventilation of patients with high tetraplegia, as well as facilitating speech, mobility, and so forth, [103].

## 8. Conclusions

SCIs often lead to restrictive respiratory changes. VC is an indicator of overall pulmonary function; patients with severely impaired VC may require assisted ventilation. It is best to proceed with intubation under controlled circumstances rather than waiting until the condition becomes an emergency. Mechanical ventilation can adversely affect the structure and function of the diaphragm. Early tracheostomy following short orotracheal intubation is probably beneficial in selected patients. Weaning should start as soon as possible, and the best modality is PVFB, due to its efficacy and ease of performance. Appropriate candidates can sometimes be freed from mechanical ventilation by electrical stimulation. The conservative management of respiratory dysfunction in SCI patients can be used to help remove secretions by various techniques and to increase ventilation by respiratory exercises and noninvasive positive air pressure support.
